# 4-Chlorothymol Exerts Antiplasmodial Activity Impeding Redox Defense System in *Plasmodium falciparum*

**DOI:** 10.3389/fphar.2021.628970

**Published:** 2021-03-10

**Authors:** Saurabh Kumar, Pooja Rani Mina, Ravi Kumar, Anirban Pal, Ateeque Ahmad, Sudeep Tandon, Mahendra P. Darokar

**Affiliations:** ^1^ Molecular Bioprospection Department, CSIR-Central Institute of Medicinal and Aromatic Plants, Lucknow, India; ^2^ Process Chemistry and Technology Department, CSIR-Central Institute of Medicinal and Aromatic Plants, Lucknow, India

**Keywords:** 4-chlorothymol, reactive oxygen species, reactive nitrogen species, reduced glutathione, glutathione reductase, glutathione S-transferase, synergy

## Abstract

Malaria remains one of the major health concerns due to the resistance of *Plasmodium* species toward the existing drugs warranting an urgent need for new antimalarials. Thymol derivatives were known to exhibit enhanced antimicrobial activities; however, no reports were found against *Plasmodium* spp. In the present study, the antiplasmodial activity of thymol derivatives was evaluated against chloroquine-sensitive (NF-54) and -resistant (K1) strains of *Plasmodium falciparum*. Among the thymol derivatives tested, 4-chlorothymol showed potential activity against sensitive and resistant strains of *P. falciparum*. 4-Chlorothymol was found to increase the reactive oxygen species and reactive nitrogen species level. Furthermore, 4-chlorothymol could perturb the redox balance by modulating the enzyme activity of GST and GR. 4-Chlorothymol also showed synergy with chloroquine against chloroquine-resistant *P. falciparum*. 4-Chlorothymol was found to significantly suppress the parasitemia and increase the mean survival time in *in vivo* assays. Interestingly, in *in vivo* assay, 4-chlorothymol in combination with chloroquine showed higher chemosuppression as well as enhanced mean survival time at a much lower concentration as compared to individual doses of chloroquine and 4-chlorothymol. These observations clearly indicate the potential use of 4-chlorothymol as an antimalarial agent, which may also be effective in combination with the existing antiplasmodial drugs against chloroquine-resistant *P. falciparum* infection. *In vitro* cytotoxicity/hemolytic assay evidently suggests that 4-chlorothymol is safe for further exploration of its therapeutic properties.

## Introduction

Malaria remains one of the major health concerns especially in tropical countries, despite the fact that numerous drugs/combinations are available against the protozoan parasite *Plasmodium* species (Pink et al., 2005). The situation is further aggravated due to the resistance of *Plasmodium* to the existing drugs.

According to the World Health Organization, about 229 million people are affected by malaria globally, resulting in around 409,000 deaths annually (WHO, 2020). Malarial drug resistance is further posing challenges as first-line combination therapy is becoming ineffective. Therefore, new strategies are required to overcome the challenges of drug resistance in malaria parasites (Fairhurst et al., 2012).

During intraerythrocytic stages, *P. falciparum* degrades hemoglobin into free heme and globins generating reactive oxygen species (ROS) in the process (Deshmukh and Trivedi, 2013). Besides, ROS is also generated into mitochondria through various metabolic processes (Trivedi et al., 2005; Van Dooren et al., 2006). In *P. falciparum*, ROS is detoxified majorly through glutathione redox system involving various enzymes such as glutathione reductase (GR), glutathione-S-transferase (GST), and glutathione peroxidase (GPx) (Müller, 2004; Müller, 2015).

Thymol (2-isopropyl-5-methylphenol) is a natural monoterpenoid phenol derivative of cymene and is extracted from *Thymus vulgaris*, *Trachyspermum ammi*, and various other plants as a white crystalline substance. It possesses several pharmacological activities such as antimicrobial, antitumor, antimutagenic, antigenotoxic, analgesic, antispasmodic, anti-inflammatory, antiparasitic, antiplatelet, and insecticidal activity (Can Baser, 2008; Chauhan and Kang, 2015). Thymol derivatives are known to exhibit enhanced activities like antifungal, antibacterial, and larvicidal activity against *Aedes aegypti* (Kaur et al., 2014; Silva et al., 2017).

The principal objective of this study was to identify thymol derivative, which is active against drug-sensitive (NF-54) and -resistant strains (K1) of *P. falciparum*. Experimental observations revealed that the most active derivative 4-chlorothymol could exert its antiplasmodial action by inducing oxidative stress in chloroquine-resistant strain of *P. falciparum* in concentration dependent manner. 4-Chlorothymol was found impeding redox defense system resulting into the accumulation of higher oxidative stress, which is known to affect macromolecules such as DNA, proteins, and lipids as well as cell organelles like mitochondria (Trachootham et al., 2008). Furthermore, the antiplasmodial activity of 4-chlorothymol could be validated in *in vivo* assays against chloroquine-resistant strain of *P. yoelii nigeriensis*, wherein significant decrease in parasitemia and increase in mean survival time were observed. 4-Chlorothymol was also observed to interact synergistically with chloroquine against chloroquine-resistant strain (K1) of *P. falciparum*.

## Material and Methods

### Reagents

Fast Blue BB salt, hypoxanthine, Triton X-100, thiobarbituric acid, trichloroacetic acid, dimethyl sulfoxide (DMSO), bovine serum albumin, 3-(4,5-dimethylthiazol-2-yl)-2,5-diphenyl bromide (MTT), phosphate-buffered saline (PBS), D-sorbitol, acridine orange, ethidium bromide, buthionine sulfoximine (BSO), glutathione disulfide, glutathione reductase, *o*-phthalaldehyde, nicotinamide adenine dinucleotide phosphate (NADPH), chloroquine diphosphate, artesunate, and clotrimazole were purchased form Sigma-Aldrich (St. Louis, MO, United States), and Albumax II, Roswell Park Memorial Institute 1,640 (RPMI-1640), Dulbecco’s modified eagle medium (DMEM), fetal bovine serum (FBS), and fungizone were purchased from Gibco (Grand Island, United States). Griess reagent kit, chloromethyl-2′,7′-dichlorodihydrofluorescein diacetate (CM-H_2_DCFDA), and SYBR Green qPCR super mix were purchased from Invitrogen (Carlsbad, CA, United States). MitoScreen Flow Cytometry kit (Cat No.551302) and APO-BrdU apoptosis detection kit (Cat No. 556381) were obtained from BD Biosciences (Franklin Lakes, NJ, United States).

### Chemistry

Synthesis and structures of thymol derivatives have been reported earlier (Kaur et al., 2014).

### Animal Ethics

*In vivo* study using Swiss albino mice was duly approved by Committee for the Purpose of Control and Supervision of Experimentation on Animals (CPCSEA), Govt. of India, through the Institutional Animal Ethics Committee (CIMAP/IAEC/2016–19/09).

### *In vitro* Antiplasmodial Activity of Thymol and Its Derivatives

The *in vitro* growth inhibition of *P. falciparum* was assessed using ring stage culture of *P. falciparum* at 1.2% parasitemia in 2% hematocrit. Culture (200 µL) was transferred to 96-well plates; test sample was added at different concentrations (0.1–100 μg/ml) and incubated for 48 h at 37°C. Artesunate and chloroquine were taken as standard drugs and culture without test sample served as unexposed (negative) control. Parasitemia was monitored by preparing thin blood smear, which was fixed in methanol and stained with Giemsa (Alin et al., 1990). Parasitemia was counted in 1,000 total erythrocytes and percent inhibition was calculated by the following equation:

$$\text{Inhibition parasitemia}\;\left(\%\right)=\frac{\text{parasitemia in control}-\text{parasitemia in test}}{\text{parasitemia in control}}\times 100.$$

IC_50_ (mean ± SEM) was determined from concentration dependent growth inhibition data by nonlinear regression analysis.

### Isolation of Parasites From Infected Erythrocytes and Preparation of Parasite Lysate

Parasite was isolated as described previously (Choubey et al., 2006). Infected erythrocytes (10%) were centrifuged at 800x g for 2 min, washed, and resuspended in cold PBS. An equal volume of 0.05% saponin was added to the erythrocyte suspension and kept on ice for 15 min. It was centrifuged at 1,300x g for 5 min to obtain the parasite pellet, and finally the pellet was washed with PBS and used immediately. Parasites lysate was obtained by mild sonication (30 s pulse, bath-type sonicator) of isolated parasite in PBS at 4°C which was stored at −20°C for future use.

### Measurement of Reactive Oxygen Species (ROS) Level

Intracellular ROS was measured using chloromethyl-2′, 7′-dichlorodihydrofluorescein diacetate (CM-H_2_DCFDA) dye that fluoresces on reaction with reactive oxygen species in cells (Kumar et al., 2008). In brief, *P. falciparum* culture of strain (K1) at 10% parasitemia was incubated in presence of different concentrations (6.52 µM, 13.04 µM, and 26.08 µM) of 4-chlorothymol and positive control (artesunate) at 0.010 µM for 24 h. After incubation, culture was washed with incomplete growth media and further incubated for 30 min in culture medium with chloromethyl-2′, 7′-dichlorodihydrofluorescein diacetate (CM-H_2_DCFDA). ROS level was determined using a spectrofluorometer (FLUOStar Omega, BMG Lab tech) at wavelengths 485 nm and 520 nm for excitation and emission, respectively. Percent increase in the level of ROS was calculated in comparison to unexposed (negative) control. Each experiment was performed in triplicate.

Intracellular ROS production was also measured by flow cytometry using the culture after 30 min incubation with chloromethyl-2′, 7′-dichlorodihydrofluorescein diacetate (CM-H_2_DCFDA) (Eruslanov and Kusmartsev, 2010). Samples were analyzed using a flow cytometer (BD Biosciences) equipped with a 488 nm argon laser as light source. Percentage of CM-H_2_DCFDA positive cells and mean fluorescence intensity were calculated using FACS Diva software (BD Biosciences).

### Measurement of Hydroxyl Radical (^•^OH) Level

For the measurement of ^•^OH level, *P. falciparum* culture of strain (K1) at 10% parasitemia was incubated in presence of different concentrations (6.52 µM, 13.04 µM, and 26.08 µM) of 4-chlorothymol and a positive control (artesunate) at 0.010 µM with 0.5% DMSO for 24 h. Intracellular ^•^OH level was determined in parasite lysate by measuring methanesulfinic acid (Kumar et al., 2008). Parasite lysate was further processed by adding toluene : butanol (3 : l), wherein methanesulfinic acid remains in the water phase due to the reaction of ^•^OH with dimethyl sulfoxide (DMSO). Methanesulfinic acid thus formed was allowed to react with Fast Blue BB salt and the intensity of resulting yellow chromophore was measured using a spectrophotometer (FLUOStar Omega, BMG Lab tech) at 425 nm. Percent increase in the level of hydroxyl radical was calculated in comparison to unexposed (negative) control. All the experiments were performed in triplicate.

### Measurement of Nitric Oxide (NO) Level

Nitrite (NO_2_^−^) level was measured using the Griess reagent kit (Invitrogen, United States, Cat. No. G7921). *P. falciparum* culture of strain (K1) at 10% parasitemia was incubated in presence of different concentrations (6.52 µM, 13.04 µM, and 26.08 µM) of 4-chlorothymol and positive control sodium-nitroprusside (SNP) for 24 h. Out of 1 ml, 300 μL cultures supernatant containing nitrite was taken in separate microcentrifuge tube. To this 260 μL of MQ water and 40 μL of Griess reagent (N-(1-naphthyl)-ethylenediamine: sulfanilic acid) were added, mixed well, and incubated for 30 min at 25°C. In parallel, a reference sample was prepared by mixing 20 μL of Griess reagent and 280 μL of MQ water. The absorbance of the samples was measured using a spectrophotometer (FLUOStar Omega, BMG Lab tech) at 548 nm (Müller, 2004). Percent increase in nitrite production was calculated in comparison to unexposed (negative) control. All the experiments were performed in triplicate.

### Measurement of Total Glutathione Level

Total glutathione level was determined as previously described (Becker et al., 2003). For the measurement of total glutathione, *P. falciparum* culture of strain (K1) at 10% parasitemia was incubated in presence of different concentrations (6.52 µM, 13.04 µM, and 26.08 µM) of 4-chlorothymol and positive control buthionine sulfoximine (BSO) at 139.48 µM for 24 h. Parasite lysate was mixed with an equal volume of 10% TCA and the protein precipitate was removed by centrifugation at 10,000 *g* for 10 min. An equal volume of 20 mM 5,5′-dithionitrobenzoic acid (dissolved in 0.8 M Tris-Cl, pH 9) was added to supernatant, with 5 µL glutathione reductase (200 U/ml) mixed well and incubated for 20 min at room temperature to yield the yellow chromophore of thionitrobenzoic acid, which was measured at 412 nm using spectrophotometer (FLUOStar Omega, BMG Lab tech). Percent decrease in total glutathione was calculated in comparison to unexposed (negative) control. All the experiments were performed in triplicate.

### Measurement of Reduced Glutathione (GSH) and Oxidized Glutathione (GSSG) Level

Reduced glutathione (GSH) and oxidized glutathione (GSSG) levels were fluorometrically determined as previously described (Hissin and Hilf, 1976). *P. falciparum* culture of strain (K1) at 10% parasitemia was incubated in presence of different concentrations (6.52 µM, 13.04 µM, and 26.08 µM) of 4-chlorothymol and positive control (artesunate) at 0.010 µM for 24 h. For GSH measurement, parasite lysate was added to the reaction mixture which contained sodium phosphate buffer (0.1 mM, 150 μL, pH 8) and *o*-phthalaldehyde (20 µL of 1 mg/ml stock in methanol) and incubated for 15 min at room temperature. GSH level was determined using a spectrofluorometer (FLUOStar Omega, BMG Lab tech) at wavelengths 365 nm and 430 nm for excitation and emission, respectively. The same method was applied for GSSG measurement, except the parasite lysate (50 µL) was first incubated with N-ethylmaleimide (NEM) for 5 min in the dark to prevent further oxidation of GSH to GSSG. Results are expressed as redox index GSH/GSSG ratio.

### Measurement of Glutathione Reductase (GR) Activity

Glutathione reductase (GR) activity was determined using previously described method (Sturm et al., 2009). *P. falciparum* culture of strain (K1) at 10% parasitemia was incubated in presence of different concentrations (6.52 µM, 13.04 µM, and 26.08 µM) of 4-chlorothymol and positive control ellagic acid at 0.67 µM for 24 h. GR activity was determined in parasite lysate using GR assay buffer (20.5 mM KH_2_PO_4_, 26.5 mM K_2_HPO_4_, 1 mM EDTA, 200 mM KCl, pH 6.9) in the presence of 100 µM (NADPH) and 1 mM glutathione disulfide as substrates. The change in absorbance per minute at 340 nm was monitored using a spectrophotometer (FLUOStar Omega, BMG Lab tech). Percent decrease in enzymatic activity of GR was calculated in comparison to unexposed (negative) control. All the experiments were performed in triplicate.

### Measurement of Glutathione-S-Transferase (GST) Activity

Glutathione-S-transferase (GST) activity was determined using a previously described method (Sturm et al., 2009). *P. falciparum* culture of strain (K1) at 10% parasitemia was incubated in presence of different concentrations (6.52 µM, 13.04 µM, and 26.08 µM) of 4-chlorothymol and positive control ellagic acid at 0.67 µM for 24 h. GST activity was determined in parasite lysate using 1.0 mM chlorodinitrobenzene (CDNB) with 1.0 mM GSH in 100 mM HEPES, 1 mM EDTA, pH 6.5. The change in absorbance per minute was monitored at 340 nm using a spectrophotometer (FLUOStar Omega, BMG Lab tech). Percent decrease in enzymatic activity of GST was calculated in comparison to unexposed (negative) control. All the experiments were performed in triplicate.

### qRT-PCR Analysis

The real time quantification of the RNA templates was analyzed by SYBR Green ER qPCR super mix (Cat No: 11,760,100, Invitrogen, United States) using 7900HT fast real time PCR system (Applied Biosystems, United States). Relative quantification was carried out by applying the ΔΔCT method (Livak and Schmittgen, 2001). 18S rRNA reference gene was taken as endogenous control for data normalization. The transcriptional profiles of glutathione reductase (*GR*) and glutathione-S-transferase (*GST*) were analyzed in *P. falciparum* culture of strain (K1) at 10% parasitemia after incubation in presence of different concentrations (6.52 µM, 13.04 µM, and 26.08 µM) of 4-chlorothymol for 24 h (Supplementary Table S1). All the experiments were performed in triplicate.

### Measurement of Mitochondrial Membrane Potential

Mitochondrial membrane potential was measured as described previously (Kumar et al., 2008). *P. falciparum* culture of strain (K1) at 10% parasitemia was incubated in presence of different concentrations (6.52 µM, 13.04 µM, and 26.08 µM) of 4-chlorothymol and positive control (carbonyl cyanide *m*-chlorophenyl hydrazone, CCCP) at 3 μM for 24 h. After incubation, culture was washed with PBS by centrifugation at 800 *g* for 2 min; JC-1 dye was added at final concentration of 5 µM and incubated in dark up to 30 min. After washing with PBS, smears were prepared for confocal microscopic (Zeiss LSM 880) examination.

Simultaneously, parasite was isolated from the culture that was exposed to 4-chlorothymol as described above. Isolated parasite was washed with PBS by centrifugation at 800 *g* for 2 min; JC-1 dye was added at final working concentration of 5 µM and incubated in dark up to 30 min. Remaining JC-1 dye was removed by washing with PBS and pellet was analyzed using flow cytometer (LSRII BD Biosciences) equipped with the 488 nm argon laser beam as light source (Rottenberg and Wu, 1998). Percentage of JC-1 green positive cells (FL1-green channel) and mean green fluorescence intensity were calculated using FACS Diva analysis software.

### DNA Fragmentation (TUNEL) Assay

DNA fragmentation was measured using an APO-BrdU apoptosis detection kit (BD Biosciences, Cat No. 556381) (Singh et al., 2015). *P. falciparum* culture of strain (K1) at 10% parasitemia was incubated in presence of different concentrations (6.52 µM, 13.04 µM, and 26.08 µM) of 4-chlorothymol and positive control (camptothecin) at 3 μM for 24 h. After incubation, parasite culture was washed with PBS and resuspended in 4% (w/v) paraformaldehyde in PBS (pH-7.4) for fixing the cells. Samples were stained with a FITC-labeled anti-BrdU mAb and analyzed using the flow cytometer (LSRII BD Biosciences) equipped with the 488 nm argon laser beam as light source. Percentage of FITC-BrdU positive cells and mean green fluorescence intensity were calculated using FACS Diva analysis software. All the experiments were performed in triplicate.

### Measurement of Lipid Peroxidation

Lipid peroxidation was quantified by the formation of malondialdehyde (MDA), a final product in the sequence of lipid peroxidation reaction. The MDA concentration was assessed with the help of thiobarbituric acid reactive species (TBARS) assay as described previously (Singh et al., 2015). *P. falciparum* culture of strain (K1) at 10% parasitemia was incubated in presence of different concentrations (6.52 µM, 13.04 µM, and 26.08 µM) of 4-chlorothymol and positive control (clotrimazole) at 0.92 µM for 24 h. Parasite lysate was mixed with 20% (v/v) trichloroacetic acid, after which the mixture was centrifuged; supernatant was mixed with 0.8% (w/v) thiobarbituric acid and incubated at 95°C for 60 min. The absorbance of the resulting solution was measured using a spectrophotometer (FLUOStar Omega, BMG Lab tech) at 532 nm, subtracting the value for nonspecific absorption at 600 nm. All the experiments were performed in triplicate.

### Protein Carbonyl Assay

Protein carbonyl was measured using assay described previously (Levine et al., 1994). *P. falciparum* culture of strain K1 at 10% parasitemia was incubated in presence of different concentrations (6.52 µM, 13.04 µM, and 26.08 µM) of 4-chlorothymol and positive control (artesunate) at 0.010 µM for 24 h. Parasite lysate was treated with 10% TCA to precipitate protein and allowed to react with DNPH (dissolved in 2 N HCl) at final concentration of 5 mM for 1 h at 37°C. After incubation, pellet was washed with ethanol : ethyl acetate (1:1) and pellet was dissolved in 6 M guanidine-hydrochloride and absorbance measured at 366 nm using a spectrophotometer (FLUOStar Omega, BMG Lab tech). All the experiments were performed in triplicate.

### *In Vivo* Antimalarial Activity

*In vivo* antimalarial activity of 4-chlorothymol and combination (4-chlorothymol and chloroquine) was evaluated using Swiss albino mice (5mice/group) against *Plasmodium yoelii nigeriensis* (chloroquine-resistant rodent malaria strain) (Khare et al., 2018). 4-Chlorothymol was suspended in 0.7% CMC (carboxy methyl cellulose), which was administered orally to the experimental animals 1 h after infection at doses 25 mg/kg, 50 mg/kg, and 100 mg/kg body weight for 7 days consecutively. The untreated control (vehicle) group animals received an equal volume of 0.7% CMC. Artesunate (positive control) was administered orally at 100 mg/kg for 4 days only, since it completely clears the parasitemia within this duration. Thin smears of blood obtained from the tail of mice were prepared every alternate day till day 28 to determine the percent of parasitemia. The blood smears were fixed in methanol, stained with Giemsa stain, and microscopically observed for parasitemia. Chemosuppression and mean survival time (MST) for each group were recorded. The hemoglobin level was also checked on day 8 (peak parasitemia) and quantified through cyanmethemoglobin (CMG) method (Moore et al., 1981). Briefly, 2 μL of blood was diluted in 0.5 ml of Drabkin’s reagent and the absorbance was read at 540 nm against blank using CMG as standard through spectrophotometer (FLUO Star Omega, BMG Lab tech). In combination, 4-chlorothymol with chloroquine at ½ ED_50_ (2.5 + 1.87 mg/kg body weight) and ED_50_ (5.0 + 3.75 mg/kg body weight) doses was administered orally to the experimental animals 1 h after infection for four consecutive days.

### Fix Ratio Combination Assay

Drug interaction studies were performed using a modification of the fixed ratios method (Fivelman et al., 2004). Initially, the 50% inhibitory concentration (IC_50_) values of the individual test compounds were determined against chloroquine-resistant *P. falciparum* (K1). Chloroquine and 4-chlorothymol were taken at a concentration which is 5 times higher than their respective IC_50_ and then mixed volume by volume at 4:1 (0.52: 2.40 μg/ml), 3:2 (0.39: 4.80 μg/ml), 1:1 (0.325: 6.0 μg/ml), 2:3 (0.260: 7.2 μg/ml), and 1:4 (0.130: 9.6 μg/ml); these ratios were further diluted with twofold dilution up to fifth well. The IC_50_ values for different combinations were determined and the fractional inhibitory concentrations (FIC_50_) were calculated using IC_50_ concentrations of individual and in combinations. All the experiments were performed in triplicate and mean value of IC_50_ was taken for FIC_50_ calculations. The interactions were assessed based on their FIC and isobologram that was plotted using FIC_50_ values obtained from each ratio.

ΣFIC <1 synergism, ≥ 1 and < 2 additive, ≥ 2 and < 4 antagonism.

### Statistical Analyses

Statistical analysis and graphical representation of data were done using GraphPad Prism version 5. Dunnett’s test was used to compare the exposed and unexposed (negative) control by one-way analysis of variance (ANOVA). *p* < 0.05 was considered statistically significant (*p < 0.05, **p < 0.01 and ***p < 0.001). Data are expressed as mean ± SEM, *n* = 5 for *in vivo* experiments and *n* = 3 for *in vitro* experiments.

## Results

### *In Vitro* Antiplasmodial Activity of Thymol and Its Derivatives

Thymol and its eight derivatives were evaluated for *in vitro* antiplasmodial activity against the chloroquine-sensitive (NF54) and -resistant (K1) strains. The most active derivative, 4-chlorothymol, showed IC_50_ value of 0.93 ± 0.24 μg/ml (5.05 µM) and 2.40 ± 0.42 μg/ml (13.04 µM) against sensitive (NF-54) and resistant (K1) strains, respectively (Table 1). IC_50_ of the clinically used drug artesunate was 0.0026 ± 0.0002 μg/ml (0.0067 µM) and 0.0039 ± 0.0003 μg/ml (0.010 µM) against NF-54 and K1 strains, respectively. Similarly, chloroquine exhibited IC_50_ values of 0.0011 ± 0.0004 μg/ml (0.0021 µM) and 0.130 ± 0.04 μg/ml (0.252 µM) against NF-54 and K1 strains, respectively. In double vital staining, decrease in parasite survival was observed after the incubation of 48 h (Supplementary Figure S1). Cytotoxicity of 4-chlorothymol was evaluated at various concentrations against Vero cell line, wherein CC_50_ value of 140.42 ± 3.42 μg/ml (760.86 µM) was observed. The selectivity index (CC_50_ against Vero cell line/IC_50_ against strain K1) for 4-chlorothymol was found to be 58, which is considered to be a good safety profile. 4-Chlorothymol at a concentration of 100 μg/ml (543.47 µM) did not exhibit the significant hemolytic activity (3.83 ± 0.42%).

**TABLE 1:** Antiplasmodial activity of thymol and its derivatives against chloroquine-sensitive (NF54) and -resistant (K1) strains of *P. falciparum*.

Molecules		IC_50_ (µg/ml)	
		NF-54	K1
1	Thymol	12.0 ± 1.40 (80 µM)	12.3 ± 0.94 (82 µM)
2	Thymol acetate	18.8 ± 1.96 (97.91 µM)	21.7 ± 1.80 (113.2 µM)
3	Thymol butyrate	5.4 ± 0.47 (24.54 µM)	6.0 ± 0.56 (27.27 µM)
4	Thymol propionate	63.0 ± 3.07 (305.82 µM)	72.0 ± 3.86 (349.51 µM)
5	Monobromothymol	30.0 ± 2.08 (131.57 µM)	34.3 ± 3.08 (150.43 µM)
6	Dibromothymol	36.6 ± 3.25 (118.83 µM)	40.2 ± 2.98 (130.51 µM)
7	4-Chlorothymol	0.93 ± 0.24 (5.05 µM)	2.40 ± 0.42 (13.04 µM)
8	Dichlorothymol	23.0 ± 1.52 (105.50 µM)	27.0 ± 2.05 (123.85 µM)
9	Trichlorothymol	48.0 ± 3.45 (189.72 µM)	52.0 ± 5.48 (205.53 µM)
10	Chloroquine	0.0011 ± 0.0004 (0.0021 µM)	0.130 ± 0.04 (0.252 µM)
11	Artesunate	0.0026 ± 0.0002 (0.0067 µM)	0.0039 ± 0.0003 (0.010 µM)
12	Ellagic acid	–	0.205 ± 0.09 (0.67 µM)
13	Clotrimazole	–	0.32 ± 0.04 (0.92 µM)
14	Buthionine sulfoximine	–	31.12 ± 4.38 (139.48 µM)

Data are expressed as mean ± SEM (*n* = 3).

### 4-Chlorothymol Increased the Intracellular ROS Level

**FIGURE 1:**
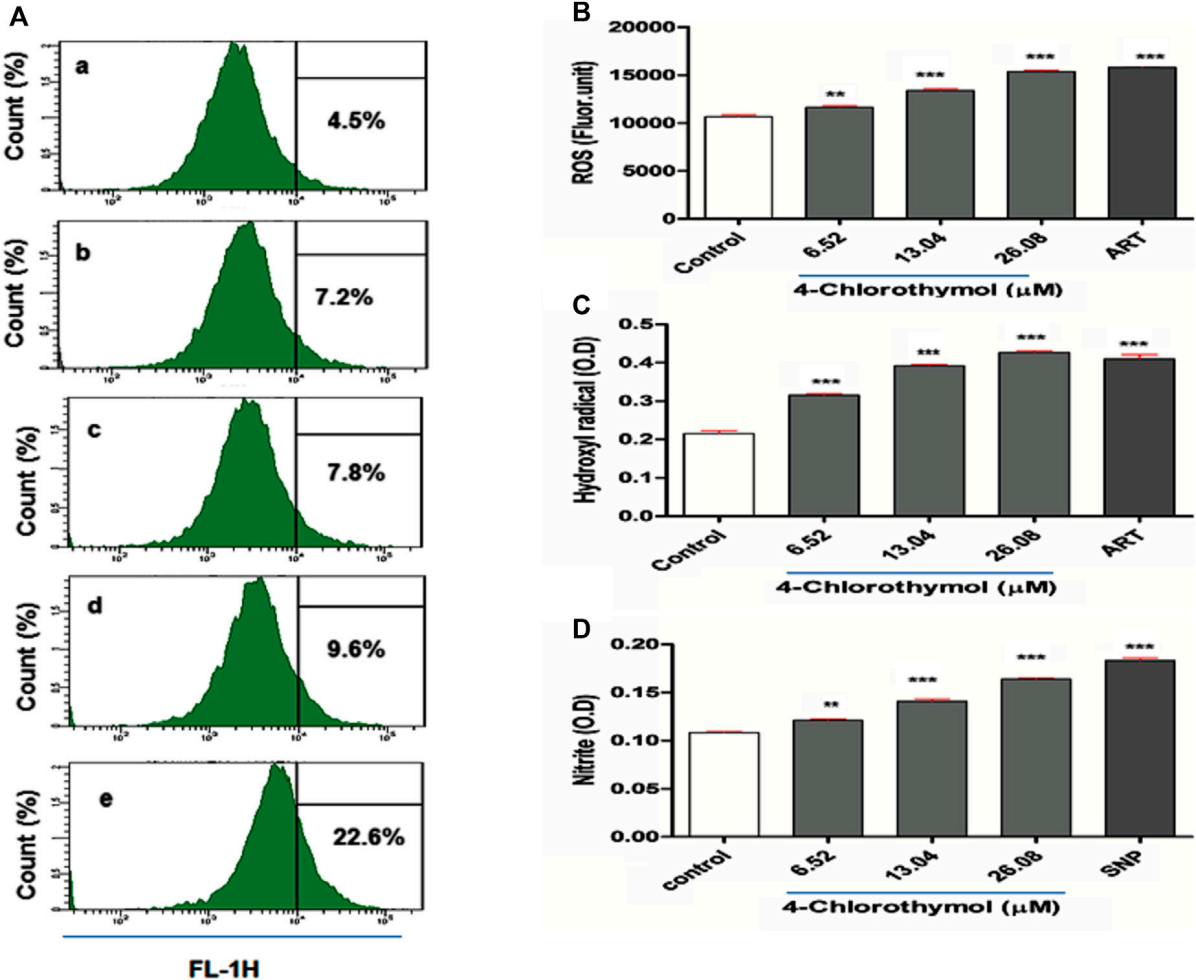
Effect of 4-chlorothymol on the generation of reactive oxygen species (ROS) and nitric oxide (NO). **(A)** Flow cytometry histograms showing increase in DCF positive cells, **(A)** unexposed culture (negative control), **(B)** 6.52 µM, **(C)** 13.04 µM, and **(D)** 26.08 µM concentrations of 4-chlorothymol and **(E)** positive control (artesunate) at 0.010 µM. **(B)** Spectrofluorometric measurement of reactive oxygen species (ROS), control = unexposed culture (negative control) and positive control (ART = artesunate). **(C)** Hydroxyl radical (^•^OH) level (OD at 425 nm), control = unexposed culture (negative control) and ART = artesunate (positive control) and **(D)** nitrite (NO_2_^−^) level (O.D at 548), control = unexposed culture (negative control) and SNP = sodium-nitroprusside (positive control). Experiments were performed thrice (*n* = 3) and data expressed as mean ± SEM. For statistical significance, Dunnett’s multiple test was performed. **p < 0.01 and ***p < 0.001 indicate a significant difference as compared to unexposed culture (negative control).

In order to evaluate the effect of 4-chlorothymol on reactive oxygen species accumulation, ROS was measured through spectrofluorometric and flow cytometric analysis. In case of flow cytometric analysis, concentration dependent increase in CM-H_2_DCFDA positive cells (7.2%, 7.8%, and 9.6% at 6.52 µM, 13.04 µM, and 26.08 µM) was observed in 4-chlorothymol exposed culture as compared to the unexposed (negative) control (4.5%). In case of positive control (artesunate) at 0.010 µM, up to 22.6% increase in CM-H_2_DCFDA positive cells was found as compared to unexposed (negative) control (Figure 1A). Similarly, in spectrofluorometric analysis, the concentration dependent increase in ROS level was observed as 9.05 ± 1.90%, 25.49 ± 2.39%, and 44.08 ± 1.66% at 6.52 µM, 13.04 µM, and 26.08 µM concentrations, respectively, as compared to unexposed (negative) control. In case of positive control (artesunate) at 0.010 µM, increased level of ROS (48.21 ± 1.22%) was observed as compared to unexposed (negative) control (Figure 1B). In presence of 4-chlorothymol dose dependent enhanced accumulation of ROS was observed which could be measured by two different methods (spectrofluorometric and flow cytometric analysis).

### 4-Chlorothymol Enhanced the Hydroxyl Radical (^•^OH) Level

The effect of 4-chlorothymol on ^•^OH level was assessed using spectrophotometric method, wherein concentration dependent increase of 45.60 ± 1.2%, 81.25 ± 2.47%, and 97.35 ± 4.80% was observed at 6.52 µM, 13.04 µM, and 26.08 µM concentrations, respectively, as compared to unexposed (negative) control. In case of positive control (artesunate) at 0.010 µM, up to 88.12 ± 3.70% increase in ^•^OH level was observed as compared to unexposed (negative) control (Figure 1C). This observation indicates the role of 4-chlorothymol in the accumulation of ^•^OH level through the interference in oxidative stress response mechanism.

### 4-Chlorothymol Increased the Reactive Nitrogen Species (RNS) Level

The effect of 4-chlorothymol on reactive nitrogen species (RNS) was evaluated through spectrophotometer in form of nitrite (NO_2_^−^) level. The concentration dependent increase in nitrite level was 11.88 ±0.58%, 30.59 ± 0.56%, and 51.41 ± 1.00% at 6.52 µM, 13.04 µM, and 26.08 µM concentrations, respectively, as compared to unexposed (negative) control. In case of positive control (sodium-nitroprusside), up to 68.37 ± 1.10% increase in nitrite (NO_2_^−^) level was observed as compared to unexposed (negative) control (Figure 1D).

### 4-Chlorothymol Hinders Total Glutathione Level

**FIGURE 2:**
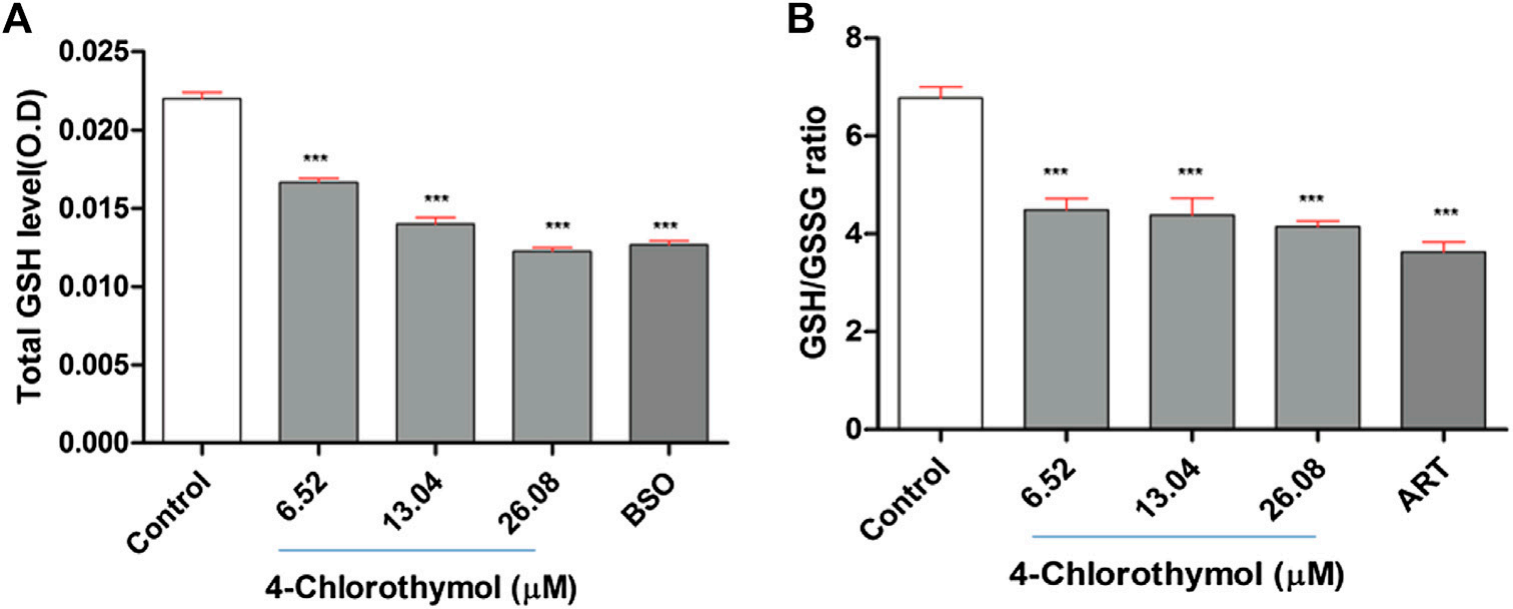
Effect of 4-chlorothymol on **(A)** total glutathione level (O.D at 412 nm), control = unexposed culture (negative control) and positive control (BSO = buthionine sulfoximine) at 139.48 μM, and **(B)** reduced (GSH) and oxidized (GSSG) glutathione ratio, control = unexposed culture (negative control) and positive control (ART = artesunate) at 0.010 μM. Experiments were performed thrice (*n* = 3) and data expressed as mean ± SEM. For statistical significance, Dunnett’s multiple test was performed. ***p < 0.001 indicates a significant difference compared with unexposed culture (negative control).

The effect of 4-chlorothymol on total glutathione level was measured through spectrophotometer. The concentration dependent decrease was observed in total glutathione level which was recorded as 24.69 ± 1.45%, 37.79 ± 2.28%, and 43.33 ±1.78% at 6.52 µM, 13.04 µM, and 26.08 µM concentrations, respectively, as compared to unexposed (negative) control. In case of positive control (buthionine sulfoximine) at 139.48 µM, up to 41.31 ± 1.98% decrease in the level of glutathione was observed as compared to unexposed (negative) control (Figure 2A). Glutathione is known for its critical role in detoxification of ROS and in presence of 4-chlorothymol, its level was found significantly decreased in dose dependent manner indicating that 4-chlorothymol plays some role in detoxification of ROS in *P. falciparum*.

### 4-Chlorothymol Decreases GSH/GSSG Ratio

The ratio between reduced glutathione and oxidized glutathione (GSH/GSSG ratio) is known to be an important biomarker of the redox balance in the cell and hence the effect of 4-chlorothymol was assessed on GSH/GSSG ratio through spectrofluorometric analysis. The concentration dependent decrease in GSH/GSSG ratio was observed to be 34.37 ± 3.10%, 36.06 ± 4.95%, and 39.39 ±1.64% at 6.52 µM, 13.04 µM, and 26.08 µM concentrations, respectively, as compared to unexposed (negative) control. In case of positive control (artesunate) at 0.010 µM, up to 46.97 ± 2.90% decrease in GSH/GSSG ratio was found as compared to unexposed (negative) control (Figure 2B). The decrease in GSH/GSSG ratio indicates the role of 4-chlorothymol in redox imbalance, thereby increasing the accumulation of oxidative stress in form of ROS and RNS level in chloroquine-resistant strain (K1) of *P. falciparum*.

### 4-Chlorothymol Decreases Enzymatic Activity of Glutathione Reductase (GR)

The glutathione reductase (GR) plays important role in glutathione mediated oxidative stress balancing by converting oxidized glutathione to its reduced form. The effect of 4-chlorothymol on glutathione reductase (GR) enzymatic activity was measured through spectrophotometric analysis. The concentration dependent decrease in GR activity was observed to be 5.35 ± 0.47 nM/mg, 1.96 ± 0.16 nM/mg, and 0.84 ± 0.16 nM/mg at 6.52 µM, 13.04 µM, and 26.08 µM concentrations, respectively, as compared to the unexposed (negative) control (39.95 ± 4.54 nM/mg). In case of positive control (ellagic acid) at 0.67 µM, decrease (0.48 ± 0.09 nM/mg) in GR enzyme activity was observed as compared to unexposed (negative) control (Table 2). Decreased GR enzyme activity in presence of 4-chlorothymol might be affecting GSH/GSSG ratio resulting into redox imbalance in form of higher accumulation of ROS/RNS.

**TABLE 2:** 4-Chlorothymol decreases the activity of redox enzymes in chloroquine-resistant strain (K1) of *P. falciparum*.

Concentrations (µM)	Glutathione reductase		Glutathione S-transferase	
	Concentration (nM/mg)	Percentage	Concentration (nM/mg)	Percentage
Negative control	39.95 ± 4.54	100	20.48 ± 1.82	100
6.52	5.35 ± 0.47**	13.39	13.67 ± 0.94*	66.74
13.04	1.96 ± 0.16**	4.90	6.67 ± 1.14***	32.56
26.08	0.84 ± 0.16**	2.10	2.27 ± 0.73***	11.08
Positive control ellagic acid (0.67)	0.48 ± 0.09**	1.20	12.57 ± 1.57**	61.37

The GR and GST contents of the controls were normalized to 100%. Data are expressed as mean ± SEM (*n* = 3). *p < 0.05, **p < 0.01, and ***p < 0.001 indicate a significant difference compared with unexposed (negative) control.

### 4-Chlorothymol Affects Enzymatic Activity of Glutathione S-Transferase (GST)

The effect of 4-chlorothymol on glutathione S-transferase (GST) enzymatic activity was measured through spectrophotometric analysis. The concentration dependent decrease in GST enzymatic activity was observed to be 13.67 ± 0.94 nM/mg, 6.67 ± 1.14 nM/mg, and 2.27 ± 0.73 nM/mg at 6.52 µM, 13.04 µM, and 26.08 µM concentrations, respectively, as compared to the unexposed (negative) control (20.48 ± 1.82 nM/mg). In case of positive control (ellagic acid) at 0.67 µM, decrease (12.57 ± 1.57 nM/mg) in GST activity as compared to unexposed (negative) control was observed (Table 2).

4-Chlorothymol was observed to inhibit the enzymatic activity of both GR and GST. However, the imbibition of GR is higher (∼95%) than that of GST (∼68%) at IC_50_ concentration of 4-chlorothymol resulting into redox imbalance (GSH/GSSG ratio). GST is also known to degrade hydrogen peroxide into water and oxygen and hence inhibition of GST in presence of 4-chlorothymol may also help in higher accumulation of ROS.

### 4-Chlorothymol Regulates Expression of Redox Enzymes

**FIGURE 3:**
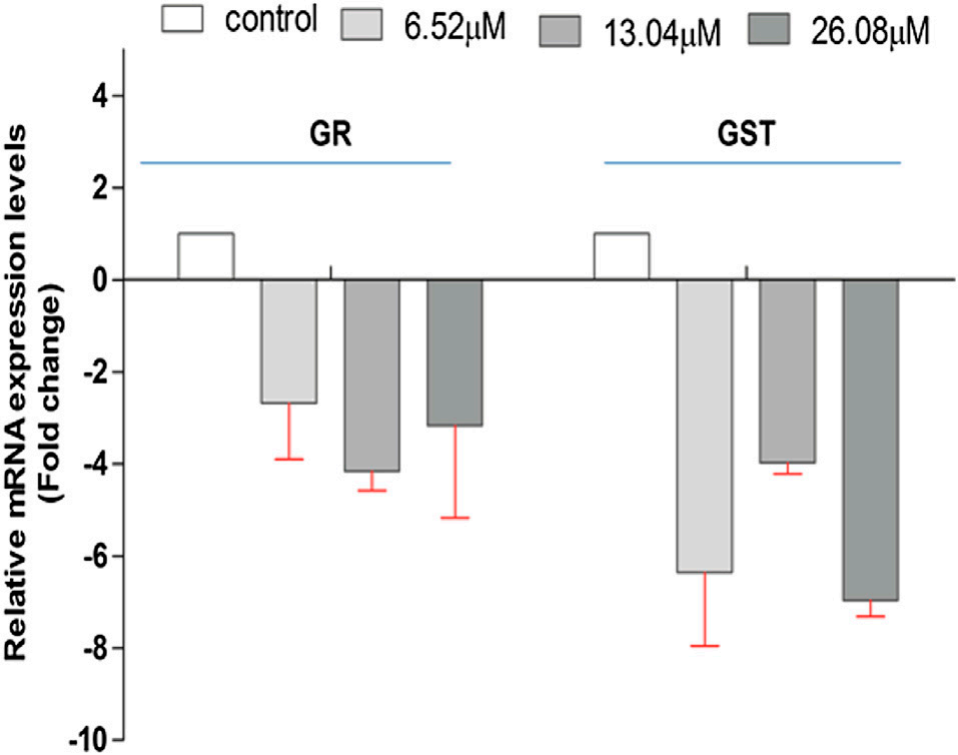
Effect of 4-chlorothymol on the relative gene expression of *GR* and *GST* in chloroquine-resistant strain (K1) of *P. falciparum* using qRT-PCR. The relative expression analysis of each gene was carried out by normalizing the data with 18S-rRNA as an endogenous control. The RQ values in *y*-axis are showing fold-changes in genes expression (mRNA) relative to the unexposed culture (negative control). GST = glutathione S-transferase and GR = glutathione reductase. Experiments were performed thrice (*n* = 3) and data expressed as mean ± SD.

The effect of 4-chlorothymol on the expression of genes encoding *GST* and *GR* that are involved in oxidative stress response in *P. falciparum* was evaluated through qRT-PCR analysis using resistant strain (K1) of *P. falciparum*. The expression of *GST* and *GR* genes was observed to be downregulated in presence of 4-chlorothymol at 6.52 µM, 13.04 µM, and 26.08 µM concentrations as compared to unexposed (negative) control (Figure 3).

### 4-Chlorothymol Induces Loss of Mitochondrial Membrane Potential

**FIGURE 4:**
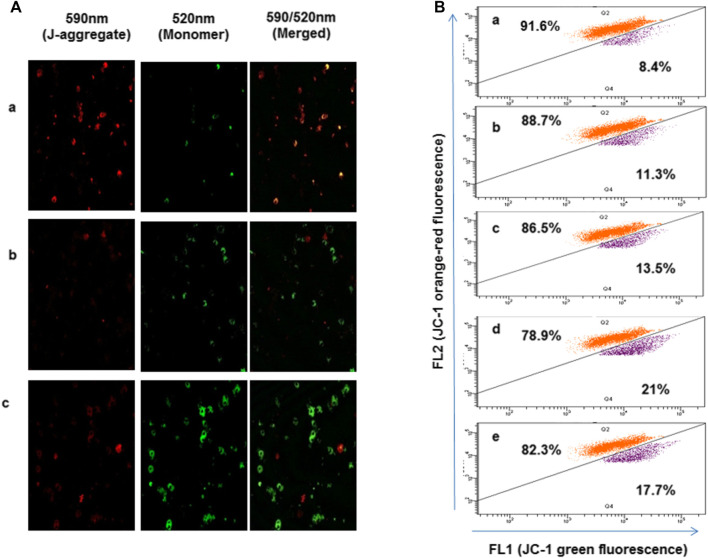
Effect of oxidative stress on mitochondrial membrane potential, **(A)** confocal microscopic analysis showing loss of mitochondrial membrane potential after exposure to 4-chlorothymol at IC_50_ concentration, **(A)** unexposed culture (negative control), **(B)** 4-chlorothymol, and **(C)** positive control (CCCP) at 3 μM. JC-1 monomer green (loss in membrane potential), JC-1 aggregates red (healthy mitochondria). **(B)** Flow cytometry histograms showing shift in the cell population toward FL1 (JC-1) green channel and increased mean green fluorescence intensity after the exposure to 4-chlorothymol at various concentrations, **(A)** unexposed culture (negative control), **(B)** 6.52 µM, **(C)** 13.04 µM, **(D)** 26.08 µM, and **(E)** positive control (CCCP) at 3 μM.

To study the effect of oxidative stress accumulated in presence of 4-chlorothymol on mitochondrial membrane potential, parasite was stained with JC-1dye and analyzed through confocal microscopy and flow cytometry. In case of confocal microscopic analysis, increase in green fluorescence as compared to unexposed (negative) control was observed in 4-chlorothymol exposed parasite culture at IC_50_ concentration. Similarly, in case of positive control (3 μM CCCP), an increase in green fluorescence as compared to unexposed (negative) control was observed (Figure 4A). In flow cytometric analysis, cells with green fluorescence (JC-1 green channel) indicate loss of membrane potential which was observed as 11.3%, 13.5%, and 21.0% at 6.52 µM, 13.04 µM, and 26.08 µM concentration of 4-chlorothymol, respectively, as compared to the unexposed (negative) control (8.4%). In case of positive control (3 μM CCCP), up to 17.7% cells with green fluorescence (JC-1 channel) as compared to unexposed (negative) control were observed (Figure 4B).

### 4-Chlorothymol Causes DNA Fragmentation in *P. falciparum*

**FIGURE 5:**
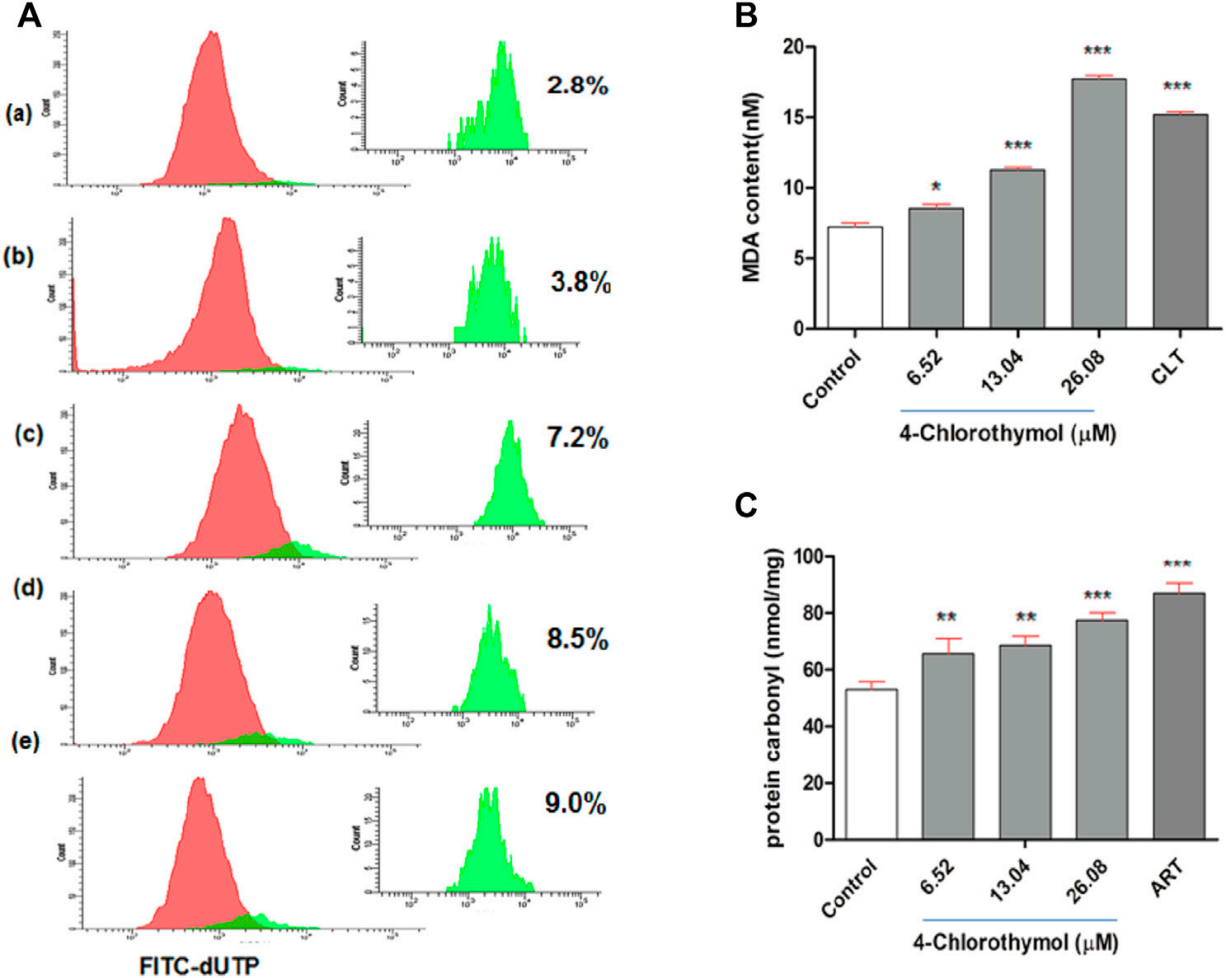
Effect of 4-chlorothymol induced oxidative stress on macromolecules. **(A)** Flow cytometry histograms showing increase in TUNEL-positive cells (highlighted in green) indicating DNA fragmentation at various concentrations of 4-chlorothymol, **(A)** unexposed culture (negative control), **(B)** 6.52 µM, **(C)** 13.04 µM, **(D)** 26.08 µM, and **(E)** positive control (camptothecin) at 3μM. **(B)** Spectrophotometric measurement of MDA content (nM), control = unexposed culture (negative control) and positive control (CLT = clotrimazole) at 0.92μM, and **(C)** spectrophotometric measurement of protein carbonyl content (nmol/mg), control = unexposed culture (negative control) and positive control (ART = artesunate) at 0.010 μM. Experiments were performed thrice (*n* = 3) and data expressed as mean ± SEM. For statistical significance, Dunnett’s multiple test was performed (*p < 0.05, **p < 0.01, and ***p < 0.001).

In order to assess the effect of oxidative stress accumulated in presence of 4-chlorothymol on the DNA fragmentation, TUNEL assay was performed using flow cytometer. It was observed that the oxidative stress induced by 4-chlorothymol at 6.52 µM, 13.04 µM, and 26.08 µM concentrations was able to increase green fluorescence intensity which is proportional to fragmented DNA. A shift in the distribution of cells toward the right side of FITC green channel was also recorded in the presence of 4-chlorothymol. The level of TUNEL-positive cells was 3.8%, 7.2%, and 8.5% at 6.52 µM, 13.04 µM, and 26.08 µM concentrations, respectively, as compared to unexposed (negative) control (2.8%). In case of positive control (3 μM camptothecin), increase in TUNEL-positive cells up to 9.0% was observed (Figure 5A).

### 4-Chlorothymol Increases Malondialdehyde Level in *P. falciparum*

In order to assess the effect of oxidative stress accumulated in presence of 4-chlorothymol on the malondialdehyde level, lipid peroxidation in terms of malondialdehyde (MDA) formation in *P. falciparum* was measured through TBARS (thiobarbituric acid reactive substances) assay. The concentration dependent increase of 8.54 ± 0.81 nM, 11.27 ± 0.50 nM, and 17.72 ± 0.70 nM was observed in MDA level at 6.52 µM, 13.04 µM, and 26.08 µM concentrations of 4-chlorothymol, respectively, as compared to unexposed (negative) control (7.22 ± 0.75 nM). In case of positive control (clotrimazole at 0.92 µM), increased (15.18 ± 0.60 nM) level of MDA was observed as compared to unexposed (negative) control (Figure 5B).

### 4-Chlorothymol Increases Protein Carbonylation in *P. falciparum*

The effect of oxidative stress accumulated in presence of 4-chlorothymol on protein carbonylation was measured spectrophotometrically. The concentration dependent increase was also observed in protein carbonyl level as 65.60 ± 5.42 nmol/mg, 68.60 ± 3.27 nmol/mg, and 77.42 ± 2.73 nmol/mg at 6.52 µM, 13.04 µM, and 26.08 µM concentrations of 4-chlorothymol, respectively, as compared to unexposed (negative) control (53.03 ± 2.73 nmol/mg). In case of positive control (artesunate at 0.010 µM), increased (86.96 ± 3.7 nmol/mg) level of protein carbonyl as compared to unexposed (negative) control was observed (Figure 5C).

### *In Vivo* Antimalarial Efficacy of 4-Chlorothymol

In order to test its *in vivo* efficacy, 4-chlorothymol was administered orally at the doses of 25 mg/kg, 50 mg/kg, and 100 mg/kg body weight to *P. yoelii nigeriensis* infected Swiss albino mice. On eighth day after infection, 89.12 ± 2.45%, 92.88 ± 1.25%, and 98.12 ± 0.61% chemosuppression were observed at 25 mg/kg, 50 mg/kg, and 100 mg/kg body weight doses, respectively, as compared to vehicle control (Table 3). Similarly, mean survival time was also found increased up to 12.43 days, 16.23 days, and 18.2 days upon the treatment of 4-chlorothymol at 25 mg/kg, 50 mg/kg, and 100 mg/kg body weight doses, respectively, as compared to vehicle control (MST 9.0 days). Interestingly, at 100 mg/kg body weight dose of 4-chlorothymol, two mice were found to survive till 28th day (Supplementary Figure S2A). The hemoglobin level in infected mice was also found increased upon the treatment of 4-chlorothymol as 5.65 ± 0.55, 6.09 ± 0.35, and 7.3 ± 0.81 g/dl at 25 mg/kg, 50 mg/kg, and 100 mg/kg body weight dose, respectively, as compared to the vehicle control (1.58 ± 0.6).

**TABLE 3:** *In vivo* antiplasmodial activity of 4-Chlorothymol using Swiss albino mice infected with *P. yoelii nigeriensis*.

Groups	Dose (mg/kg)	^#^Percent parasitemia			MST (days)	^#^Percent chemosuppression at day 8	^#^Percent increased hemoglobin
		4 Days 6 Days 8 Days					
Vehicle control	–	2.07 ± 0.50	9.86 ± 2.78	30.90 ± 4.89	9.0	–	–
4-Chlorothymol	25	0.92 ± 0.21	1.83 ± 0.68	3.36 ± 0.90^***^	12.43	89.12 ± 2.45	257.59
	50	0.29 ± 0.09	1.19 ± 0.52	2.20 ± 0.88^***^	16.23	92.88 ± 1.25	285.44
	100	0.14 ± 0.06	0.32 ± 0.08	0.58 ± 0.35^***^	18.2	98.12 ± 0.61	362.0
Artesunate (positive control)	100	0.00	0.00	0.00^***^	˃28	100 ± 0.00	488.60

MST = mean survival time. ^#^Results are represented as mean percentage ±SEM (*n* = 5), ***p < 0.001 indicates a significant difference compared with vehicle control on day eighth.

When 4-chlorothymol and chloroquine were administered orally in combination at their respective ½ ED_50_ (2.5 + 1.87 mg/kg body weight) and ED_50_ (5.0 + 3.75 mg/kg body weight) doses, 96.11 ± 0.69% and 97.67 ± 0.42% chemosuppression were observed as compared to vehicle control. Similarly, MST was also found increased up to 16.68 days and 17.6 days at ½ ED_50_ and ED_50_ doses, respectively, as compared to vehicle control (MST 9.1 days). In case of a group of mice treated with 4-chlorothymol at ½ ED_50_ (2.5 mg/kg body weight) and ED_50_ (5 mg/kg body weight) doses, increased mean survival time of 13.26 days and 14.84 days was observed, whereas chloroquine at ED_50_ (3.75 mg/kg body weight) dose exhibited MST of 14.74 days (Supplementary Figure S2B). In case of combination (4-chlorothymol and chloroquine), dose dependent increase in hemoglobin level of 8.80 ± 0.0.43 g/dl and 12.25 ± 0.49 g/dl was observed at their respective ½ ED_50_ (2.5 + 1.87 mg/kg body weight) and ED_50_ (5.0 + 3.75 mg/kg body weight) doses (Table 4). Hemoglobin level in vehicle control (untreated infected) mice was comparatively very low (1.13 ± 0.15 g/dl).

**TABLE 4:** *In vivo* antiplasmodial activity of combination using Swiss albino mice infected with *P. yoelii nigeriensis*.

Groups	Dose (mg/kg)	^#^Percent parasitemia			MST (Days)	^#^Percent chemosuppression at day 8	^#^Percent increased hemoglobin
		4 Days 6 Days 8 Days					
Vehicle control	–	1.62 ± 0.31	29.77 ± 1.32	70.3 ± 1.15	9.1	–	–
4-Chlorothymol	2.5	0.08 ± 0.04	21.02 ± 1.06	49.79 ± 3.82^***^	13.26	29.17 ± 2.0	189.28
	5	0.02 ± 0.011	17.38 ± 1.18	31.49 ± 1.80^***^	14.84	55.20 ± 3.12	457.14
Chloroquine	3.75	0.01 ± 0.001	6.42 ± 0.93	29.25 ± 1.83^***^	14.74	58.39 ± 3.16	561.83
4-Chlorothymol + chloroquine	2.5 + 1.87	0.00	0.06 ± 0.004	2.73 ± 0.40^***^	16.68	96.11 ± 0.69	673.18
	5 + 3.75	0.00	0.017 ± 0.005	1.63 ± 0.24^***^	17.6	97.67 ± 0.42	976.32
Artesunate (positive control)	100	0.00	0.00	0.00^***^	˃28	100 ± 00	886.44

MST = mean survival time. ^#^Results are represented as mean percentage ±SEM (*n* = 5), ***p < 0.001 indicates a significant difference compared with vehicle control on day eighth.

### *In Vitro* Interaction Between 4-Chlorothymol and Chloroquine

**FIGURE 6:**
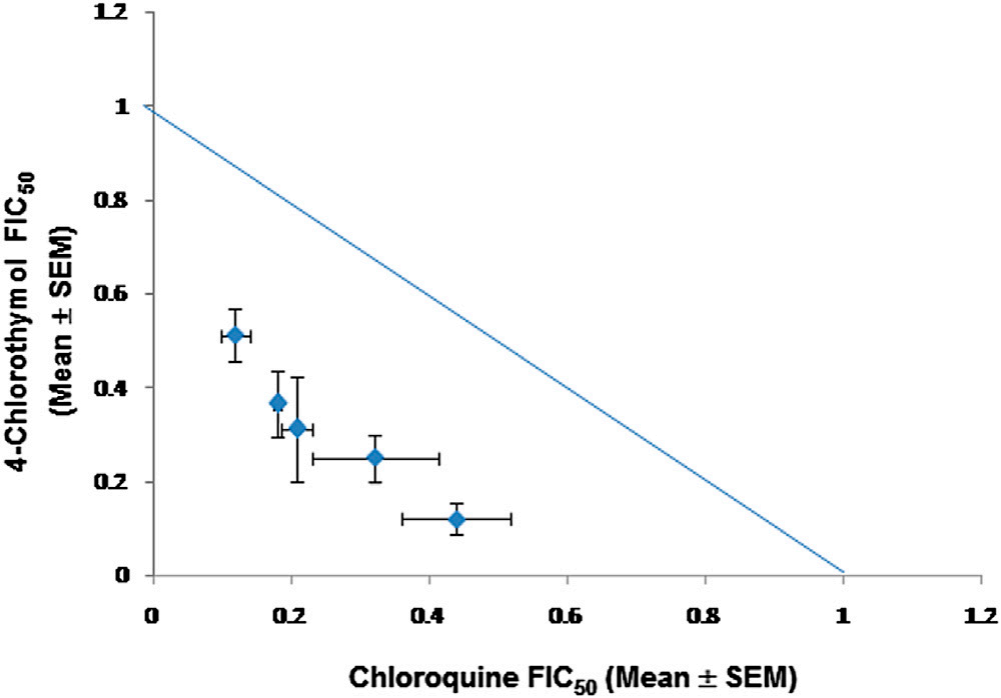
Isobologram representing interaction between 4-chlorothymol and chloroquine against drug-resistant strain (K1) of *P. falciparum*. Mean FIC_50_ of 4-chlorothymol and chloroquine were taken on the *y*- and *x*-axes, respectively. Experiments were performed thrice (*n* = 3) and data expressed as mean ± SEM. Sum of mean FIC_50_ values on the line shows additive, above the line shows antagonistic, and below the line shows synergistic interaction.

When 4-chlorothymol was evaluated in combination with chloroquine, it showed synergistic interaction at different ratios (Table 5; Figure 6). The sum of FIC_50_ of chloroquine and 4-chlorothymol in the combination was observed to be less than one (1.0), indicating the synergy between the two combination partners.

**TABLE 5:** *In vitro* interaction of 4-chlorothymol with chloroquine at different concentrations against chloroquine-resistant strain (K1) of *P. falciparum*.

Fix ratio	*IC_50_ (µg/ml)		*FIC_50_		ƩFIC_50_	^#^Interactions
	Chloroquine	4-Chlorothymol	Chloroquine	4-Chlorothymol		
5:0	0.130 ± 0.04 (0.252 µM)	–	–	–	–	–
4:1	0.057 ± 0.010 (0.110 µM)	0.287 ± 0.078 (1.559 µM)	0.438 ± 0.078	0.119 ± 0.032	0.558	Synergistic
3:2	0.041 ± 0.011 (0.079 µM)	0.600 ± 0.119 (3.260 µM)	0.320 ± 0.091	0.250 ± 0.049	0.570	Synergistic
1:1	0.027 ± 0.002 (0.052 µM)	0.745 ± 0.268 (4.048 µM)	0.207 ± 0.022	0.310 ± 0.111	0.518	Synergistic
2:3	0.023 ± 0.008 (0.044 µM)	0.875 ± 0.166 (4.755 µM)	0.179 ± 0.006	0.364 ± 0.069	0.544	Synergistic
1:4	0.015 ± 0.002 (0.029 µM)	1.225 ± 0.132 (6.657 µM)	0.117 ± 0.020	0.510 ± 0.055	0.628	Synergistic
0:5	–	2.40 ± 0.42 (13.043 µM)	–	–	–	–

FIC_50_ = IC_50_ in combination/IC_50_ of drug alone and ƩFIC_50_ = FIC_50_ values of chloroquine + FIC_50_ values of 4-chlorothymol at different ratio. *IC_50_ and *FIC_50_ expressed as mean ± SEM (n = 3), and ^#^Σ FIC <1 synergism, Σ FIC ≥1 and <2 additive, Σ FIC ≥2 and <4 antagonism.

## Discussion

Malaria is one of the most distressing parasitic diseases despite the availability of numerous drugs acting against the protozoan parasite *Plasmodium* spp. in its human host. However, development of resistance renders most of the existing drugs ineffective. Several strategies are being utilized to combat these parasitic diseases including derivatives approaches (Flannery et al., 2017) and natural remedies (Willcox and Bodeker, 2004).

In the present study, among eight derivatives of thymol, 4-chlorothymol was found to be the most active against chloroquine sensitive (NF-54) as well as resistant strains (K1) of *P. falciparum*. In a previous study, among different halogenated tropane 3α-(2′-chlorobenzoyloxy) derivatives with chloro group it was found to be the most active (Cretton et al., 2014). Chloro group is also known to increase the lipophilicity of molecules that improve membrane permeability (Gerebtzoff et al., 2004).

*P. falciparum* employs two systems to maintain the intracellular redox balance, i.e., glutathione/glutathione reductase and thioredoxin/thioredoxin reductase. However, glutathione mediated redox management is most prevalent (Becker et al., 1994; Atamna and Ginsburg, 1997; Meierjohann et al., 2002). In digestive vacuole, parasite uses hemoglobin for survival; in this process hemoglobin is broken down through methemoglobin intermediate into free heme which is transported to cytoplasm. Since free heme is toxic to parasite, it is either polymerized into nontoxic hemozoin or degraded into bilirubin and carbon monoxide (Gozzelino et al., 2010). Free heme degradation occurs mainly involving reduced glutathione (Ginsburg and Golenser, 2003). In hemoglobin digestion process, superoxide ions (O_2_^•^^−^) are released which are further converted into hydrogen peroxide (H_2_O_2_) by superoxide dismutase (SOD), the first enzyme of antioxidant defense system. Mitochondria are especially sensitive to oxidative stress and reduced GSH levels can affect the function of mitochondria as a result of oxidative damage due to superoxide ions (O_2_^•^^−^) that are generated during metabolic processes like electron transport system (respiratory chain) (Vega-Rodriguez et al., 2009), which are further converted into H_2_O_2_ by superoxide dismutase (SOD).

Hydrogen peroxide thus generated is further converted into hydroxyl radical (^•^OH) and hydroxyl ions (^**-**^OH) through Fenton reaction, which is known to specifically affect lipids (Atamna and Ginsburg, 1993; Ryter and Tyrrell, 2000). Hydrogen peroxide is detoxified through glutathione based redox system, wherein enzymes like glutathione reductase (GR), glutathione S-transferase (GST), and glutathione peroxidase (GPx) play a major role (Lehane et al., 2012).

4-Chlorothymol was found to increase reactive oxygen species (ROS) and reactive nitrogen species (RNS) level in concentration dependent manner. Similarly, it could also increase the level of hydroxyl radical (^•^OH). Further, 4-chlorothymol was observed to decrease the total glutathione and reduce glutathione (GSH) levels in concentration dependent manner.

In order to understand the underlying mechanism of decreased level of GSH, effect of 4-chlorothymol on the enzymatic activity of glutathione reductase and glutathione-S-transferase was evaluated. The activity of both these enzymes was found to decrease indicating the role of 4-chlorothymol in management of glutathione mediated redox balancing in *P. falciparum.* The increased level of GSSG may be attributed to comparatively less inhibition of GST as compared to GR. This observation is further supported by decreased GSH/GSSG ratio as well as downregulation of the genes encoding GR and GST in the presence of 4-chlorothymol.

In order to study the effect of oxidative stress induced by 4-chlorothymol on cellular organelles and macromolecules, various experimental evidence was recorded. The loss in mitochondrial membrane potential was observed in presence of 4-chlorothymol as evident from the confocal microscopic and flow cytometry analysis using JC-1 dye. Similarly, increase in DNA fragmentation, lipid peroxidation, and protein carbonylation was observed indicating the damage of DNA, lipids, and proteins due to oxidative stress induced by 4-chlorothymol.

Antiplasmodial activity of 4-chlorothymol could be validated using *P. yoelii nigeriensis* (chloroquine-resistant) strain infected Swiss albino mice. A significant decrease in parasitemia and increase in hemoglobin level and mean survival time were observed upon the treatment of 4-chlorothymol. Interestingly, at 100 mg/kg body weight dose of 4-chlorothymol, two mice were found to survive up to 28th day after infection. Further, in case of combination, significantly higher (97.67%) chemosuppression was observed at ED_50_ combination concentrations. Combination was able to increase the hemoglobin (12.25 ± 0.49 g/dl) level and mean survival time (17.6 days). Moreover, a significant reduction in parasitemia level on 8^th^day after infection was observed. Interestingly, in case of combination, two and three mice at ½ ED_50_ and ED_50_ body weight doses were found to survive up to the 28th day after infection.

4-Chlorothymol also showed potential in combination therapy exhibiting synergistic interaction with chloroquine which is evident from the isobologram generated based on fraction inhibitory (FIC_50_) concentration. It is well known fact that, in chloroquine-resistant strains of *P. falciparum*, elevated level of GSH is maintained, which effectively counters the oxidative stress (Davioud-Charve et al., 2001). 4-Chlorothymol was able to decrease the reduced GSH level in chloroquine-resistant strain (K1), which might be the reason for synergistic interaction between 4-chlorothymol and chloroquine.

In summary, 4-chlorothymol was found effective against both chloroquine-sensitive and -resistant strains by exerting its antiplasmodial action by accumulating oxidative stress in a concentration dependent manner. 4-Chlorothymol was found to perturb almost all aspects of redox balance, as shown by the use of various assays such as measurement of redox species, hydroxyl radical, nitric oxide level, total glutathione, reduced glutathione/oxidized glutathione ratio, and glutathione reductase/glutathione-s-transferase activity. Redox perturbation by 4-chlorothymol was also observed to affect/disrupt the function of many organelles/cellular function as shown by disruption in mitochondrial membrane potential, DNA TUNEL assay, lipid peroxidation, and protein carbonyl assay. Most importantly, 4-chlorothymol was found be active *in vivo* in murine malaria model. All these results point to future exploration of 4-chlorothymol as potential antimalarial compound.

## Conclusion

It is evident from the experimental observations that 4-chlorothymol interferes with glutathione mediated oxidative stress detoxification in chloroquine-resistant strain (K1) of *P. falciparum*, thereby increasing the level of ROS and RNS which affects different cellular processes. These observations clearly indicate the potential use of 4-chlorothymol as an antimalarial agent, which may also be effective in combination with the existing antiplasmodial drugs against chloroquine-resistant *P. falciparum* infection. *In vitro* cytotoxicity/hemolytic assay evidently suggests that 4-chlorothymol is safe for further exploration of its therapeutic properties.

## Data Availability

The raw data supporting the conclusion of this article will be made available by the authors, without undue reservation.

## References

[B1] Alin M. H. Björkman A. Ashton M. (1990). *In vitro* activity of artemisinin, its derivatives, and pyronaridine against different strains of *Plasmodium falciparum* . Trans. R. Soc. Trop. Med. Hyg. 84, 635–637. 10.1016/0035-9203(90)90129-3 2278058

[B2] Atamna H. Ginsburg H. (1993). Origin of reactive oxygen species in erythrocytes infected with *Plasmodium falciparum* . Mol. Biochem. Parasitol. 61, 231–241. 10.1016/0166-6851(93)90069-a 8264727

[B3] Atamna H. Ginsburg H. (1997). The malaria parasite supplies glutathione to its host cell--investigation of glutathione transport and metabolism in human erythrocytes infected with Plasmodium falciparum. Eur. J. Biochem. 250, 670–679. 10.1111/j.1432-1033.1997.00670.x 9461289

[B4] Becker K. Gui M. Traxler A. Kirsten C. Schirmer R. H. (1994). Redox processes in malaria and other parasitic diseases. Determination of intracellular glutathione. Histochemistry 102, 389–395. 10.1007/BF00268910 7868369

[B5] Becker K. Rahlfs S. Nickel C. Schirmer R. H. (2003). Glutathione–functions and metabolism in the malarial parasite Plasmodium falciparum. Biol. Chem. 384, 551–566. 10.1515/BC.2003.063 12751785

[B6] Can Baser K. H. (2008). Biological and pharmacological activities of carvacrol and carvacrol bearing essential oils. Curr. Pharmaceut. Des. 14, 3106–3119. 10.2174/138161208786404227 19075694

[B7] Chauhan A. K. Kang S. C. (2015). Therapeutic potential and mechanism of thymol action against ethanol-induced gastric mucosal injury in rat model. Alcohol 49, 739–745. 10.1016/j.alcohol.2015.08.004 26493110

[B8] Choubey V. Guha M. Maity P. Kumar S. Raghunandan R. Maulik P. R. (2006). Molecular characterization and localization of *Plasmodium falciparum* choline kinase. Biochim. Biophys. Acta 1760, 1027–1038. 10.1016/j.bbagen.2006.03.003 16626864

[B9] Cretton S. Bartholomeusz T. A. Mehl F. Allenbach Y. Matheeussen A. Cos P. (2014). Synthesis and *in vitro* evaluation of tropane halogenated-derivatives against malaria, sleeping sickness, Chagas disease and leishmaniasis. Med. Chem. 10, 753–758. 10.2174/1573406410666140507095430 24813684

[B10] Davioud-Charvet E. Delarue S. Biot C. Schwöbel B. Boehme C. C. Müssigbrodt A. (2001). A prodrug form of a *Plasmodium falciparum* glutathione reductase inhibitor conjugated with a 4-anilinoquinoline. J. Med. Chem. 44, 4268–4276. 10.1021/jm010268g 11708927

[B11] Deshmukh R. Trivedi V. (2013). Pro-stimulatory role of methemoglobin in inflammation through hemin oxidation and polymerization. Inflamm. Allergy—Drug Targets 12, 68–78. 10.2174/1871528111312010010 23441992

[B12] Eruslanov E. Kusmartsev S. (2010). Identification of ROS using oxidized DCFDA and flow-cytometry. Methods Mol. Biol. 594, 57–72. 10.1007/978-1-60761-411-1_4 20072909

[B13] Fairhurst R. M. Nayyar G. M. Breman J. G. Hallett R. Vennerstrom J. L. Duong S. (2012). Artemisinin-resistant malaria: research challenges, opportunities, and public health implications. Am. J. Trop. Med. Hyg. 87, 231–241. 10.4269/ajtmh.2012.12-0025 22855752 PMC3414557

[B14] Fivelman Q. L. Adagu I. S. Warhurst D. C. (2004). Modified fixed-ratio isobologram method for studying *in vitro* interactions between atovaquone and proguanil or dihydroartemisinin against drug-resistant strains of *Plasmodium falciparum* . Antimicrob. Agents Chemother. 48, 4097–4102. 10.1128/AAC.48.11.4097-4102.2004 15504827 PMC525430

[B15] Flannery E. L. Chatterjee A. K. Winzeler E. A. (2017). Antimalarial drug discovery—approaches and progress towards new medicines. Nat. Rev. Microbiol. 15, 572–862. 10.1038/nrmicro.2017.88 28736448

[B16] Gerebtzoff G. Li-Blatter X. Fischer H. Frentzel A. Seelig A. (2004). Halogenation of drugs enhances membrane binding and permeation. Chembiochem. 5, 676–684. 10.1002/cbic.200400017 15122640

[B17] Ginsburg H. Golenser J. (2003). Glutathione is involved in the antimalarial action of chloroquine and its modulation affects drug sensitivity of human and murine species of plasmodium. Redox Rep. 8, 276–279. 10.1179/135100003225002907 14962364

[B18] Gozzelino R. Jeney V. Soares M. P. (2010). Mechanisms of cell protection by heme oxygenase-1. Annu. Rev. Pharmacol. Toxicol. 50, 323–354. 10.1146/annurev.pharmtox.010909.105600 20055707

[B19] Hissin P. J. Hilf R. (1976). A fluorometric method for determination of oxidized and reduced glutathione in tissues. Anal. Biochem. 74, 214–226. 10.1016/0003-2697(76)90326-2 962076

[B20] Kaur R. Darokar M. P. Chattopadhyay S. K. Krishna V. Ahmad A. (2014). Synthesis of halogenated derivatives of thymol and their antimicrobial activities. Med. Chem. Res. 23, 2212–2217. 10.1007/s00044-013-0809-8

[B21] Khare S. Gupta M. Cheema H. S. Maurya A. K. Rout P. Darokar M. P. (2018). *Rosa damascena* restrains *Plasmodium falciparum* progression *in vitro* and impedes malaria pathogenesis in murine model. Biomed. Pharmacother. 97, 1654–1662. 10.1016/j.biopha.2017.11.130 29793328

[B22] Kumar S. Guha M. Choubey V. Maity P. Srivastava K. Puri S. K. (2008). Bilirubin inhibits *Plasmodium falciparum* growth through the generation of reactive oxygen species. Free Radic. Biol. Med. 44, 602–613. 10.1016/j.freeradbiomed.2007.10.057 18070610

[B23] Lehane A. M. McDevitt C. A. Kirk K. Fidock D. A. (2012). Degrees of chloroquine resistance in Plasmodium—is the redox system involved? Int J Parasitol Drugs Drug Resist 2, 47–57. 10.1016/j.ijpddr.2011.11.001 22773965 PMC3388501

[B24] Levine R. L. Williams J. A. Stadtman E. R. Shacter E. (1994). Carbonyl assays for determination of oxidatively modified proteins. Methods Enzymol. 233, 346–357. 10.1016/s0076-6879(94)33040-9 8015469

[B25] Livak K. J. Schmittgen T. D. (2001). Analysis of relative gene expression data using real-time quantitative PCR and the 2(-Delta Delta C(T)) method. Methods 25, 402–408. 10.1006/meth.2001.1262 11846609

[B26] Meierjohann S. Walter R. D. Müller S. (2002). Regulation of intracellular glutathione levels in erythrocytes infected with chloroquine-sensitive and chloroquine-resistant *Plasmodium falciparum* . Biochem. J. 368, 761–768. 10.1042/BJ20020962 12225291 PMC1223037

[B27] Moore G. L. Ledford M. E. Merydith A. (1981). A micromodification of the Drabkin hemoglobin assay for measuring plasma hemoglobin in the range of 5 to 2000 mg/dl. Biochem. Med. 26, 167–173. 10.1016/0006-2944(81)90043-0 7317038

[B28] Müller S. (2004). Redox and antioxidant systems of the malaria parasite *Plasmodium falciparum* . Mol. Microbiol. 53, 1291–1305. 10.1111/j.1365-2958.2004.04257.x 15387810

[B29] Müller S. (2015). Role and regulation of glutathione metabolism in *Plasmodium falciparum* . Molecules 20, 10511–10534. 10.3390/molecules200610511 26060916 PMC6272303

[B30] Pink R. Hudson A. Mouriès M. A. Bendig M. (2005). Opportunities and challenges in antiparasitic drug discovery. Nat. Rev. Drug Discov. 4, 727–740. 10.1038/nrd1824 16138106

[B31] Rottenberg H. Wu S. (1998). Quantitative assay by flow cytometry of the mitochondrial membrane potential in intact cells. Biochim. Biophys. Acta 1404, 393–404. 10.1016/s0167-4889(98)00088-3 9739168

[B32] Ryter S. W. Tyrrell R. M. (2000). The heme synthesis and degradation pathways: role in oxidant sensitivity. Heme oxygenase has both pro- and antioxidant properties. Free Radic. Biol. Med. 28, 289–309. 10.1016/s0891-5849(99)00223-3 11281297

[B33] Silva V. B. Travassos D. L. Nepel A. Barison A. Costa E. V. Scotti L. (2017). Synthesis and chemometrics of thymol and carvacrol derivatives as larvicides against *Aedes aegypti* . J Arthropod Borne Dis 11, 315–330. 29062856 PMC5641620

[B34] Singh V. Pal A. Darokar M. P. (2015). A polyphenolic flavonoid glabridin: oxidative stress response in multidrug-resistant *Staphylococcus aureus* . Free Radic. Biol. Med. 87, 48–57. 10.1016/j.freeradbiomed.2015.06.016 26117328

[B35] Sturm N. Hu Y. Zimmermann H. Fritz-Wolf K. Wittlin S. Rahlfs S. (2009). Compounds structurally related to ellagic acid show improved antiplasmodial activity. Antimicrob. Agents Chemother. 53, 622–630. 10.1128/AAC.00544-08 19015351 PMC2630624

[B36] Trachootham D. Lu W. Ogasawara M. A. Nilsa R. D. Huang P. (2008). Redox regulation of cell survival. Antioxidants Redox Signal. 10, 1343–1374. 10.1089/ars.2007.1957 PMC2932530 18522489

[B37] Trivedi V. Chand P. Srivastava K. Puri S. K. Maulik P. R. Bandyopadhyay U. (2005). Clotrimazole inhibits hemoperoxidase of Plasmodium falciparum and induces oxidative stress. Proposed antimalarial mechanism of clotrimazole. J. Biol. Chem. 280, 41129–41136. 10.1074/jbc.M501563200 15863504

[B38] Van Dooren G. G. Stimmler L. M. McFadden G. I. (2006). Metabolic maps and functions of the Plasmodium mitochondrion. FEMS Microbiol. Rev. 30, 596–630. 10.1111/j.1574-6976.2006.00027.x 16774588

[B39] Vega-Rodríguez J. Franke-Fayard B. Dinglasan R. R. Janse C. J. Pastrana-Mena R. Waters A. P. (2009). The glutathione biosynthetic pathway of Plasmodium is essential for mosquito transmission. PLoS Pathog. 5, e1000302. 10.1371/journal.ppat.1000302 19229315 PMC2636896

[B40] WHO (2020). World malaria report Geneva: world health organization., https://www.who.int/teams/global-malaria-programme/reports/world-malaria-report-2020

[B41] Willcox M. L. Bodeker G. (2004). Traditional herbal medicines for malaria. BMJ 329, 1156–1159. 10.1136/bmj.329.7475.1156 15539672 PMC527695

