# Supplementary material for: 4-Chlorothymol Exerts Antiplasmodial Activity Impeding Redox Defense System in Plasmodium falciparum
Source: Front Pharmacol. 2021 Mar 10;12:628970. doi: 10.3389/fphar.2021.628970 (PMC7988344; doi:10.3389/fphar.2021.628970)
Supplement: Supplementary file 1 [file datasheet1.doc]

**Supplementary data**

# 4-Chlorothymol exerts anti-plasmodial activity impeding redox defence system in *Plasmodium falciparum*

**Saurabh Kumar^1^, Pooja Rani Mina^1^, Ravi Kumar^1^, Anirban Pal^1^, Ateeque Ahmad^2^, Sudeep Tandon^2^, Mahendra P. Darokar^1*^**

^1^. Molecular Bioprospection Department, CSIR-Central Institute of Medicinal and Aromatic Plants, Lucknow-226015, India.

^2^. Process Chemistry and Technology Department, CSIR-Central Institute of Medicinal and Aromatic Plants, Lucknow-226015, India.

**^*^Address of Corresponding author:**

^*^**Mahendra P. Darokar**

Molecular Bioprospection Department, CSIR-Central Institute of Medicinal and Aromatic Plants (Council of Scientific and Industrial Research), P.O. CIMAP, Lucknow-226 015, India, and Tel: +91-522-2718532; Fax: +91-522-2342666

Email: [mpdarokar@yahoo.com](mailto:mpdarokar@yahoo.com), [mp.darokar@cimap.res.in](mailto:mp.darokar@cimap.res.in)

**MATERIAL AND METHODS**

***In vitro Plasmodium falciparum* culture**

The Chloroquine sensitive and resistant *P. falciparum* strains (NF-54 and K1) were maintained in human O^+^ red blood cells using RPMI-1640 medium supplemented with 25 mM HEPES, 0.2% NaHCO_3_, 370μM hypoxanthine, 40μg/ml gentamycin, 0.25μg/ml fungizone and 0.5% Albumax II at 37°C, 5% CO_2_ using the method described previously (Trager and Jensen,1976).Culture medium was changed after every 24 hrs and routinely monitored through Geimsa staining of thin blood smears. The culture was synchronized by 5% D-sorbitol treatment to obtain ring-stage parasites (Lambros and Vanderberg,1979).

***In vitro cell* cytotoxicity**

Cytotoxicity of 4-Chlorothymol was assessed using 3-(4,5-dimethylthiazol-2-yl)-2,5-diphenyl bromide (MTT) assay and expressed as CC_50_ following the method previously described (Woerdenbag et al.,1993). Cytotoxicity was carried out using Vero cell line (VERO C1008; ATCC CRL-1586). Vero cell line was incubated in presence of different concentrations of 4-Chlorothymol and positive control (chloroquine) for 48 hr. The absorbance of control and treated wells was recorded using a spectrophotometer (FLUOStar Omega, BMG Lab tech) at 570 nm. The percent inhibition was calculated by the following equation


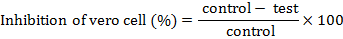


CC_50_ (mean ± SEM) was determined from concentration dependent growth inhibition data by nonlinear regression analysis. The experiments were performed in triplicate.

**Determination of selectivity index ratio (SI)**

Selectivity index (SI) is used as parameter of clinical significance. Generally, selectivity index > 2.0 is considered as safe for natural products. SI of test sample was calculated from the following equation as described previously (Sisodia et al.,2012).


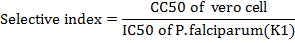


**Heamolytic activity**

The heamolytic assay was performed as described previously (Sutera et al.,1972). Briefly, 1000 μl of 10% (v/v) RBCs suspension was incubated under agitation at room temperature containing 50 μl of 4-Chlorothymol at 1-100 μg/ml concentration respectively. Triton X-100 1% (v/v) was used as a positive control and PBS (pH 7.4) as a negative control. The mixtures were then centrifuged at room temperature for 5 minutes at 8,000x g and absorbance of the supernatants was measured using a spectrophotometer (FLUOStar Omega, BMG Lab tech) at 540 nm. Percentage of RBC lysis was determined as follows


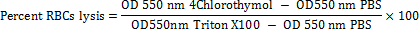


The experiments were performed in triplicate

**Double vital staining**

The Double vital staining assay was performed as described previously (Nogueira et al.,2010). *P. falciparum* culture of strain K1 at 1.5% parasitemia was incubated in presence of different concentrations of 4-Chlorothymol for 48 hr. Confocal microscopic (Zeiss LSM 880) observation of acridine orange (AO) and ethidium bromide (EtBr) double stained parasite culture smears were recorded under 515 nm wavelength, filter at 100 x magnification and image was captured for further analysis. Live cells stained with AO emit green fluorescence and dead cells stained with EtBr produce orange fluorescence.

**RESULTS**


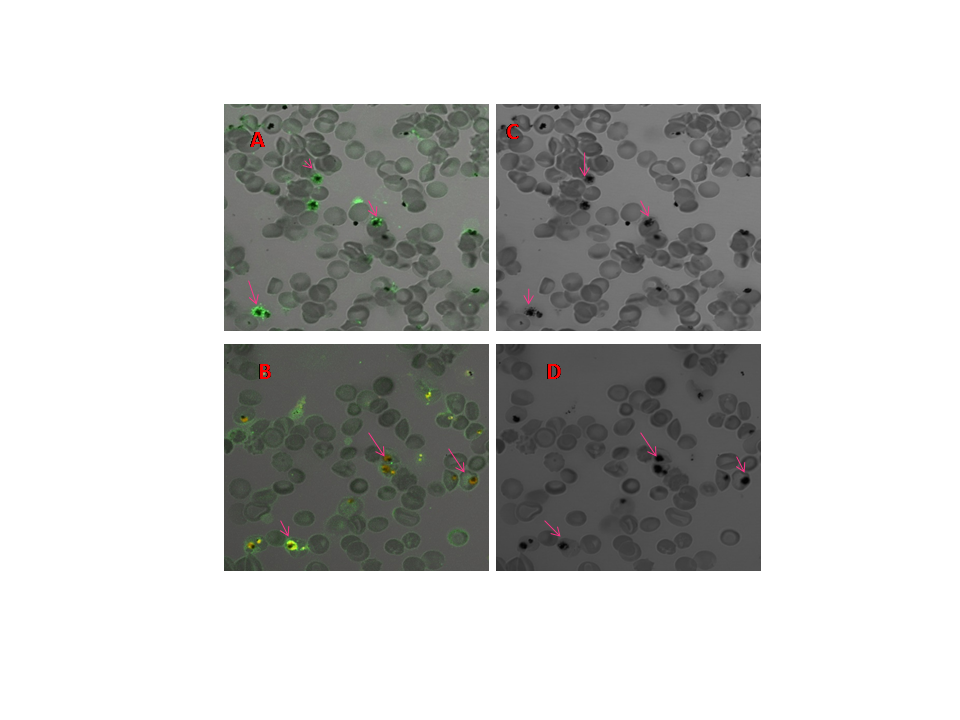


**Figure S1|** Double vital staining of *P. falciparum (*K1) after 48 hrs. Fluorescence image (A) untreated control; (B) 4-Chlorothymol; (C) and (D) corresponding differential interference contrast (DIC) image. Dead parasites emit orange fluorescence and live parasites shown green fluorescence.


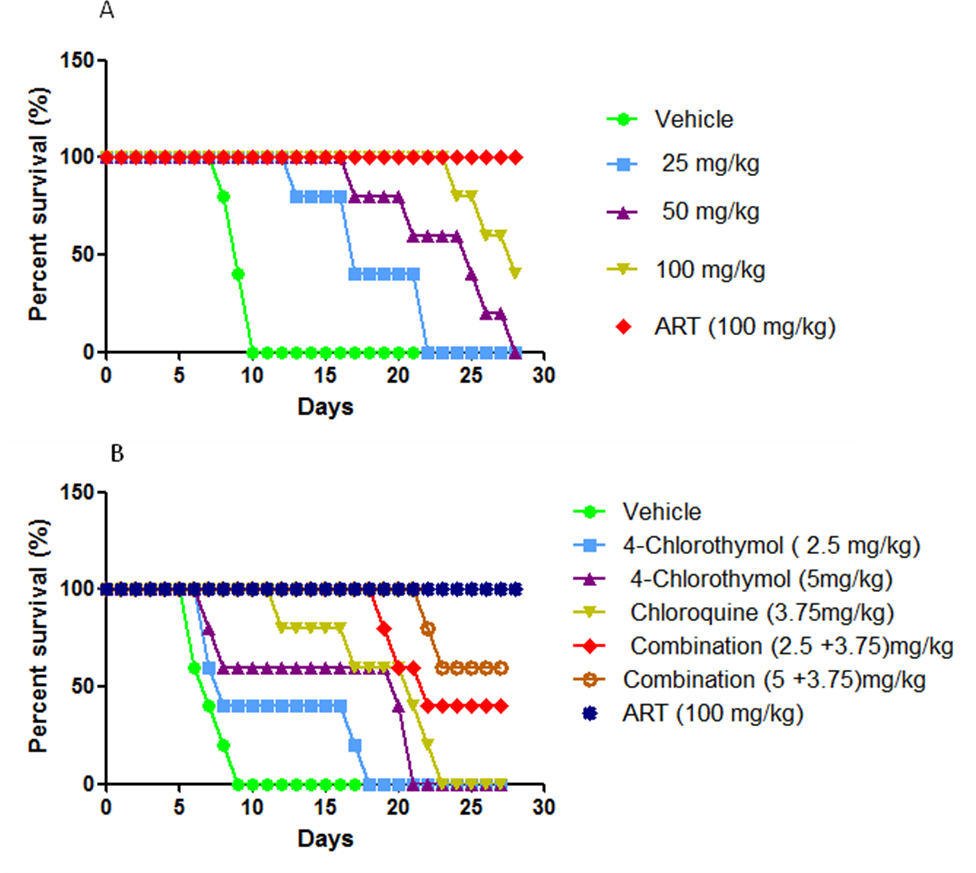


**Figure S2|**. (A) *In vivo* efficacy of 4-Chlorothymol survival kinetic upon the treatment of 4-Chlorothymol at 25 mg/kg, 50 mg/kg, and 100 mg/kg body weight as compared to vehicle and positive control (artesunate); (B) *In vivo* efficacy of 4-Chlorothymol survival kinetic upon the treatment of 4-Chlorothymol alone and in combination with chloroquine as compared to vehicle and positive control (artesunate). (ART= artesunate, vehicle= untreated (negative control).

**Table S1| Oligonucleotide primers for genes studied by quantitative real-time PCR**

| Gene name | Gene ID | Sense | Antisense |
| --- | --- | --- | --- |
| 18S rRNA | PF3D7_ 1148600 | AGGCTGCACGCGTGCTACAC | ACAATTCATCATATCTTTCAATCGG |
| GST | PF3D7_1419300 | TGATGCAAGGGGTAAAGCTGA | TGGGTACTTGCTCAAAAGGAGT |
| GR | PF3D7_1419800 | ATTGGTGGTGGAAGTGGAGC | CACACGTTCCACCTAAACGG |

**Reference**

[1] Trager W, Jensen JB. (1976). Human malaria parasites in continuous culture. Science. 193:673-5. doi: 10.1126/science.781840

[2] Lambros C, Vanderberg JP. (1979). Synchronization of *Plasmodium falciparum* erythrocytic stages in culture. J Parasitol. 65:418-20.

[3] Woerdenbag HJ, Moskal TA, Pras N, Malingré TM, el-Feraly FS, Kampinga HH, Konings AW. (1993). Cytotoxicity of artemisinin-related endoperoxides to Ehrlich ascites tumor cells. J Nat Prod. 56:849-56. doi: 10.1021/np50096a007

[4] Sisodia BS, Negi AS, Darokar MP, Dwivedi UN, Khanuja SP. (2012). Antiplasmodial activity of steroidal chalcones: evaluation of their effect on hemozoin synthesis and the new permeation pathway of *Plasmodium falciparum*-infected erythrocyte membrane. Chem Biol Drug Des. 79: 610-5.

doi: 10.1111/j.1747-0285.2012. 01323.x

[5] Sutera SP, Croce PA, Mehrjardi M. (1972). Haemolysis and subhemolytic alterations of human RBC induced by turbulent shear flow. Trans Am Soc Artif Intern Organs.18:335-41, 347. doi: 10.1097/00002480-197201000-00084

[6] Nogueira F, Diez A, Radfar A, Pérez-Benavente S, do Rosario VE, Puyet A, et al. (2010). Early transcriptional response to chloroquine of the *Plasmodium falciparum* antioxidant defence in sensitive and resistant clones. Acta Trop. 114:109-15. doi: 10.1016/j.actatropica.2010.01.013
